# Investigating epigenetic consequences of early-life adversity: some methodological considerations

**DOI:** 10.3402/ejpt.v7.31593

**Published:** 2016-11-08

**Authors:** Laura M. Fiori, Gustavo Turecki

**Affiliations:** McGill Group for Suicide Studies, Douglas Mental Health University Institute, McGill University, Montreal, Québec, Canada

**Keywords:** DNA methylation, epigenetics, early-life adversity, childhood abuse, stress

## Abstract

Stressful and traumatic events occurring during early childhood have been consistently associated with the development of psychiatric disorders later in life. This relationship may be mediated in part by epigenetic mechanisms, such as DNA methylation, which are influenced by the early-life environment. Epigenetic patterns can have lifelong effects on gene expression and on the functioning of biological processes relevant to stress reactivity and psychopathology. Optimization of epigenetic research activity necessitates a discussion surrounding the methodologies used for DNA methylation analysis, selection of tissue sources, and timing of psychological and biological assessments. Recent studies related to early-life adversity and methylation, including both candidate gene and epigenome-wide association studies, have drawn from the variety of available techniques to generate interesting data in the field. Further discussion is warranted to address the limitations inherent to this field of research, along with future directions for epigenetic studies of adversity-related psychopathology.

**Highlights of the article:**

- We identified issues regarding sample characteristics in epigenetic studies of early life adversity.
- We compared methods and technologies used for candidate gene analysis and whole epigenome studies.
- We discussed future perspectives, including combining multiple forms of large-scale data and newer technologies.

The experience of stressful and traumatic experiences, especially early in life, can lead to the development of psychiatric disorders in vulnerable individuals (Turecki, Ota, Belangero, Jackowski, & Kaufman, 2014). Psychiatric disorders are common and often chronic conditions that are associated with significant disability and mortality (Moussavi et al., 2007). Through longstanding efforts to gain insight into the etiology of these disorders, it has become clear that their development and pathology result from an intricate interaction between clinical, social, genetic, and environmental factors (Turecki & Brent, 2015). Thus, understanding the complex interplay between genes and the environment represents a key frontier in elucidating the molecular mechanisms underlying the development of psychiatric disorders (Caspi & Moffitt, 2006).

Individuals who have experienced early-life adversity (ELA), such as childhood abuse, parental neglect, and prenatal adversity, display increased rates of anxiety, depression, substance use disorders, and suicidal behaviors (Gilbert et al., 2009; McLaughlin et al., 2010; Monk, Spicer, & Champagne, 2012). Anxious and impulsive/aggressive traits are also more frequently observed among those who have experienced childhood abuse (Wanner, Vitaro, Tremblay, & Turecki, 2012) and evidence links these traits to increased risk of suicide (Brent et al., 2015; McGirr et al., 2009). Changes resulting from adverse experiences in childhood may confer characteristics that could increase chances of survival (e.g., increased anxiety), but that become maladaptive as the environment changes throughout the lifespan.

For example, physiological responses to stress, in particular, the hypothalamus–pituitary–adrenal (HPA) axis, which is responsible for controlling cortisol release and response, have been extensively shown to be dysregulated by ELA (Heim, Shugart, Craighead, & Nemeroff, 2010). Both animal and human data suggest that environmental effects on stress response are partially mediated through epigenetic regulation of gene function (McGowan et al., 2009; Weaver et al., 2004), and that such epigenetically-altered stress responses are linked to the development of psychopathologies (Turecki, 2014; Turecki & Meaney, 2014; Turecki et al., 2014). Epigenetic modifications are a collection of chemical and physical alterations in the genome that regulate the activity of genes (Tsankova, Renthal, Kumar, & Nestler, 2007). This review focuses on DNA methylation, which is the most studied and best characterized epigenetic mark. DNA methylation involves the addition of a methyl group to cytosine nucleotides, primarily at cytosine-guanine dinucleotides (CpG). DNA methylation in gene regulatory regions (i.e., promoters or enhancers) is often associated with gene repression through the recruitment of methyl-binding proteins that lead to chromatin condensation or interference with transcription factor binding (Klose & Bird, 2006).

A thorough discussion of findings linking ELA-induced epigenetic changes and psychiatric disorders is beyond the scope of this review, and studies reporting these effects have been extensively reviewed elsewhere (e.g., Jawahar, Murgatroyd, Harrison, & Baune, 2015; Lutz, Almeida, Fiori, & Turecki, 2015; Turecki et al., 2014). Comparing results across studies has, however, become increasingly difficult due to the diversity of study designs and rapidly evolving techniques for quantifying DNA methylation. We therefore focus on methodological issues that must be considered when planning and interpreting studies investigating the relationships between DNA methylation and ELA. We first discuss sample characteristics in terms of populations being examined, measures used to assess ELA, and selection of biological tissues. Second, we provide an overview of molecular techniques that can be and have been used to examine DNA methylation.

## Sample characteristics

### Sample size

Studies in behavioral epigenetics have used highly variable sample sizes, ranging from 24 (Suderman et al., 2012) to 939 (Van Der Knaap et al., 2015), with groups typically dichotomized into ELA-exposed and non-exposed controls. While some studies have used exclusively male or female subjects, most have included individuals of both genders with matched control groups. Sex-specific epigenetic effects have been suggested to occur in animal models of adversity (Kundakovic, Lim, Gudsnuk, & Champagne, 2013), and in humans there appear to be sex-specific psychiatric outcomes of ELA (Davis & Pfaff, 2014); however, there is currently insufficient evidence to conclude as to the role of sex in ELA-induced epigenetic changes in humans. In many cases, subjects with reported histories of ELA were participants in larger epidemiological cohorts, which may introduce bias when comparing studies.

### Clinical populations

Epigenetic studies performed in specific clinical populations may aid to determine how childhood adversity predisposes individuals to develop psychopathology. For example, epigenetic changes have been consistently reported in individuals with mood and anxiety disorders, eating disorders, substance use disorders, and personality disorders who had histories of childhood abuse (Turecki, 2014; Turecki et al., 2014). These studies have contributed to our understanding of the development of psychopathologies by identifying genes of interest and physiological circuits that may be affected by ELA, with the most well-studied being the HPA axis and associated genes including the glucocorticoid receptor (NRC31) and FK506 binding protein 5 (FKBP5).

### Assessment of early-life adversity

There has been substantial variability in the methods used for evaluating both the experience and severity of adversity. Many studies rely on self-reported experiences assessed through a small number of items, typically yes/no questions regarding specific forms of abuse. Although this information is useful for dichotomizing subjects, it does not account for important variables that may act as moderators, such as frequency and severity of the abuse, identity of the abuser, age, etc., which have demonstrable effects on ensuing psychopathology (Brezo et al., 2007; Fergusson, Woodward, & Horwood, 2000; Lopez-Castroman et al., 2012, 2013). Results from yes/no questionnaires may therefore be insufficient to allow investigation of the relationship between abuse and other measures. Standardized questionnaires improve the capacity to compare results between studies and provide more detailed information regarding the forms and severity of abuse. The two most frequently used questionnaires are the Childhood Experience of Care and Abuse (CECA) questionnaire (Bifulco, Brown, & Harris, 1994) and the shorter, self-administered Childhood Trauma Questionnaire (CTQ) (Bernstein et al., 1994). The CECA is an interview-based questionnaire that assesses various dimensions of the parent–child relationship during childhood and provides information regarding physical, sexual, and emotional abuse, as well as emotional and physical neglect. An important consideration is that even with standardized questionnaires, assessment of ELA is nearly always done retrospectively, and is subject to certain biases, with childhood abuse likely to be underreported (false-negatives) due to repression of memories and feelings of shame and guilt experienced by the victim, among other factors, with limited evidence of over-reporting (false-positives) (Hardt & Rutter, 2004). In addition to experiences of abuse and neglect, adversity may also be characterized by experiences of stressful family environments, including financial hardship, domestic violence, parental psychopathology, social disruption (such as adoption and war), or prenatal events, each of which can be assessed through a variety of measures (e.g., census data, police reports, and self-reports).

### Selection of age groups to examine

Studies of ELA have been performed in cohorts of children, adolescents, and adults. These groups differ in several key aspects: most importantly, the temporal relationships between the experience and assessment of ELA and DNA methylation. These differences are important to consider for several reasons. Firstly, the length of time since childhood may affect recall of the experience. Secondly, the initial epigenetic changes may induce cascades of altered methylation patterns, with gene expression differences at early stages resulting in epigenetic changes later on. Therefore, performing studies in adults may yield different results than in children or adolescents, even if the initial effects were identical (Heim, Mayberg, Mletzko, Nemeroff, & Pruessner, 2013).

The developmental stage of the abuse victim at the time of the adversity may define the nature and extent of neurobiological alterations. Abuse occurring earlier in life may have greater influence on DNA methylation, suggesting the presence of critical or sensitive periods for the epigenetic effects of ELA (Guintivano & Kaminsky, 2016; Heim & Binder, 2012). Finally, it remains unclear whether neurobiological alterations are specific to different forms of maltreatment (Heim et al., 2013), or if they are due to the psychological impact of adversity, irrespective of the type of abuse. As we increasingly recognize the importance of epigenetics, researchers have begun designing longitudinal studies with epigenetic assessments at multiple time points, which can give insight into how these modifications vary over time.

### Selection of tissues to analyze

Although the brain is the most biologically relevant tissue for psychological studies, obtaining high-quality samples is challenging and results may be influenced by factors such as post-mortem interval (Rhein et al., 2015). Studies involving living subjects must therefore rely upon peripheral samples. The three most commonly collected are blood, saliva, and buccal cells. These tissues may be examined directly, or in the case of blood, lymphoblast cell lines may be generated to facilitate downstream functional studies. There is evidence that methylation patterns vary between the brain and peripheral tissues, between regions of the brain (Davies et al., 2012; Xin et al., 2010), and between cell types in a given tissue (Mellen, Ayata, Dewell, Kriaucionis, & Heintz, 2012), adding to the complexity of analyzing differentially methylated regions from various tissue sources. Thus the selection of tissue to examine for epigenetic studies can have a considerable impact on the results and on the ability to interpret these findings.

Despite these limitations, studies using peripheral tissues are informative and are of particular relevance in the pursuit of biomarkers of disease, and in some cases have successfully mirrored results obtained using brain tissue (Turecki & Meaney, 2014). Comparison of methylation signatures across different organs and individuals have shown DNA methylation variance to be more closely linked to tissue-specificity than to individual-specificity (Schultz et al., 2015). The majority of methylation in the genome is fairly stable, with only approximately 20% of autosomal CpG sites displaying dynamic methylation between cell types and across tissues (Ziller et al., 2013). Nonetheless, while significant, overall correlation of DNA methylation in blood and brain is low (Walton et al., 2015), and subgenetic locations such as 5’UTR, gene body, 3’UTR, and CpG islands (>200 nucleotide regions with high percentages of CG dinucleotides) and shores show strikingly different levels of correlation of methylation levels between blood and brain (Hannon, Lunnon, Schalkwyk, & Mill, 2015). Therefore, variations in DNA methylation in blood do not necessarily capture variations in brain tissues.

While data from peripheral tissues may not be as informative as that obtained from the brain, there is evidence that methylation patterns in blood may be appropriate as a proxy (Tylee, Kawaguchi, & Glatt, 2013), and two conceptual frameworks have been formulated. In the “signature” model, an event contributing to disease causes specific methylation changes in blood. In the “mirror” model, similar disease-associated methylation changes occur at given genomic sites across both blood and brain tissues. A recent study investigated this concept in mice undergoing antipsychotic treatments and found support for the two hypotheses (Aberg et al., 2013).

Given the importance of tissue-specificity, DNA must be extracted from identical and well-identified tissues for all subjects. Technical advances have improved accuracy of such tissue selection, with improved isolation of specific cell types using fluorescence-assisted cell sorting, laser microdissection, and immunomagnetic separation. This has been performed in the brain to separate neuronal and non-neuronal cells (Kozlenkov et al., 2014; Labonte, Suderman, et al., 2012) and in the blood to isolate specific white blood cell types (Wang et al., 2012). Bioinformatic approaches may also be used to generate cell-type specific profiles (“deconvolution”) from mixed samples (Guintivano, Aryee, & Kaminsky, 2013; Houseman et al., 2012). An important confounder to these analyses is the potential to misinterpret results when cell-type composition is influenced by disease state (e.g., inflammation) (Mill & Heijmans, 2013). Adequate controls are needed to address this concern, and single-cell analyses may be an important approach to distinguish between the relative contributions of cell types to observed methylation changes.

## Molecular techniques

Investigations of epigenetic changes associated with ELA can be globally divided into two types: candidate gene studies and genome-wide studies. Candidate studies tend to be simpler to carry out, since choosing genes of interest at the outset limits the number of sites to evaluate for epigenetic changes. However, the last few years have seen a rise in the number and type of genome-wide approaches which, although more laborious and expensive, are less biased and have the potential to identify new genes of interest for further investigation.

### Candidate gene studies

Studies investigating DNA methylation have used a variety of molecular techniques, and these have evolved since the initial publications in behavioral epigenetics. Each technique has its own relative advantages and drawbacks, with two key differences being the size of the genomic region examined and resolution at the sequence-level. Initial studies focused on particular genes of interest, with targeted analyses to identify the effects of ELA on genes previously demonstrated to be involved in stress response or behavior. Consistent alterations in DNA methylation have been found in NR3C1 (Labonte, Yerko, et al., 2012; McGowan et al., 2009; Perroud et al., 2011), FKBP5 (Klengel et al., 2013; Tyrka et al., 2015; Yehuda et al., 2015), the serotonin transporter (SLC6A4) (Beach, Brody, Todorov, Gunter, & Philibert, 2010; Wang et al., 2012; Wankerl et al., 2014), and brain derived neurotrophic factor (Perroud et al., 2013; Thaler et al., 2014).

*Clone sequencing* is the “gold-standard” method to examine DNA methylation and involves bisulfite conversion of DNA, a chemical process that specifically converts unmethylated cytosines to uracil residues, followed by amplification and cloning of a small (less than 600 nucleotides) region of the genome. Multiple clones are sequenced for each sample, providing information on each cytosine residue in the region and indicating strand-specific patterns of methylation (i.e., which sites tend to be methylated concurrently). Although this method provides the greatest resolution, it is labor-intensive and only feasible for small genomic regions. It is thus reserved for hypothesis-driven studies (i.e., McGowan et al., 2009) or for validation of data obtained by high-throughput approaches.

*Targeted next-generation bisulfite sequencing* has begun to replace clone sequencing as the gold standard in epigenetic studies. Similar to clone sequencing, this method involves bisulfite conversion and amplification of small genomic regions, but uses next-generation sequencing (NGS) technologies to obtain sequence-resolution information for a much larger number of amplified fragments (>100) than is typically examined in clone sequencing. This method can therefore yield a much more accurate quantification of average methylation at a given site.

*Pyrosequencing* generates information regarding the specific quantities of each nucleotide at each base within a sequence, and when performed on bisulfite-converted DNA, indicates the percentage of methylation at each cytosine within the sequence being examined. The affordability of pyrosequencing, along with its accuracy and speed, has made it an attractive alternative to clone sequencing for both targeted investigations (Klengel et al., 2013; Tyrka et al., 2015) and validation studies (Suderman et al., 2014). However, pyrosequencing has the relative disadvantages of having increased sensitivity to poor-quality DNA, being more susceptible to errors in regions of repeated nucleotides, requiring physically-sensitive machinery, and only being useful to examine a relatively small number of CpG sites.

*Quantitative PCR-based methods*, such as high-resolution melt assays, are another alternative to clone sequencing. In the case of high-resolution melting, specific nucleotide composition affects the melting point of the double-stranded DNA fragment being analyzed and can be compared with non-bisulfite-treated DNA to infer the level of methylation. While this technique has been successfully applied to samples of relevance in stress studies (Perroud et al., 2013), it presents some challenges linked to the cost of labeled primers, lengthy optimization protocols and ambiguity in results interpretation, as strand- and locus-specificity are obscured in the readout.

*Mass-spectrometry-based analysis*, such as EpiTYPER (Sequenom), is a widely used technique in methylation studies (Beach et al., 2010; Melas et al., 2013). It has many advantages over other techniques, such as low cost, near single-base resolution, and sequence reads up to 600 nucleotides, and it can be outsourced to genome centers. Its main disadvantage is its inability to independently analyze two CpGs located in close proximity, which limits analysis to CpG-poor regions.

### Whole- and partial-methylome studies

Although candidate gene studies have provided important insight into the role of specific genes and methylation marks in psychiatric disorders, it has become increasingly clear that epigenetic reprogramming in response to environmental adversity occurs on a much larger scale and that genome-wide patterns of altered methylation may be more relevant than quantifying levels at specific CpG sites. Furthermore, candidate gene studies fail to assess relationships between altered methylation sites across different biological pathways and genomic regions.

*Next-generation sequencing* techniques have been adapted for use in epigenetics, with the ability to generate vast amounts of base-resolution information. NGS techniques generate the richest information, as they associate methylation marks with genomic location, strand specificity, and local sequence variations. In addition to whole-genome bisulfite sequencing (WGBS), a less costly alternative is reduced representation bisulfite sequencing, which primarily targets CpG-rich regions. Both approaches produce large sets of data but also bring specific challenges, notably the need for robust and validated bioinformatic pipelines, statistical correction for multiple testing, and validation of findings with other technological platforms and/or additional cohorts. So far, no studies investigating ELA have been published using whole-genome bisulfite or reduced representation bisulfite NGS data to date, but ongoing studies are applying WGBS to study methylation changes associated with ELA (Turecki, unpublished.

*Affinity-based approaches* are an economical alternative for methylation analysis. Methylation arrays, which involve hybridization of DNA against pre-selected sequences, allow for large-scale investigations of the epigenome and rely on an initial bisulfite-treatment of the DNA. To date, the most commonly used array has been the Illumina Human 450K array (Khulan et al., 2014; Prados et al., 2015; Suderman et al., 2015), which targets the majority of known coding genes, as well as additional known and putative regulatory regions outside of gene bodies. The use of a commercial array allows results to be compared across studies using the same technology but may not provide sufficient information on specific targets that are of interest to the researcher. One alternative is the use of methylation-specific immunoprecipitation techniques using antibodies directed against either methylated DNA (MeDIP) or against proteins known to bind methylated CpGs (e.g., MDB-IP) (Weber et al., 2005). DNA fragments can then be quantified using custom-designed microarray chips to assess methylation at specific functional (Labonte, Yerko, et al., 2012) or genomic (Suderman et al., 2012; Zhang, Wang, Kranzler, Zhao, & Gelernter, 2013) regions.

### Additional molecular considerations

The relationship between CpG methylation and gene expression has been best characterized for sites that are in regulatory regions in or near a gene body, often found in CpG islands. Accordingly, these regions have been the primary site of investigation for candidate gene studies, and follow-up functional analyses. However, whole-genome studies of many diseases have found that dynamically methylated disease-associated regions are frequently located far from transcriptional start sites, outside of CpG islands (Ziller et al., 2013). More complicated molecular techniques will be required to elucidate the downstream effects of altered methylation at these sites.

Furthermore, methylation of non-canonical CpG sites is being recognized as a key regulator of cellular function, particularly in neuronal cells (Guo et al., 2014). Preclinical evidence suggests that DNA methylation in non-CG contexts, which seems to rapidly and selectively accumulate in neuronal cells during the first few years of life (Lister et al., 2013), may be particularly sensitive to ELA.

Oxidative forms of cytosine methylation, including hydroxymethylcytosine, formylcytosine, and carboxylcytosine, which have been identified in the brain (Lunnon et al., 2016; Massart et al., 2014), are indistinguishable by many of the technologies described above and require tailored approaches to distinguish their effects from those of traditional cytosine methylation (Plongthongkum, Diep, & Zhang, 2014).

## Perspectives

Multiple technical approaches have provided evidence to support the concept that altered DNA methylation represents one of the molecular mechanisms through which ELA influences the expression of pathology-associated genes throughout the lifespan. As we have highlighted herein, researchers must carefully consider their sample characteristics in terms of psychological assessment, biological tissue, and age groups, as well as the molecular methodology they use when assessing methylation at either the gene or whole-genome level. Ultimately, each of these factors influences our ability to detect and interpret the developmental trajectory of epigenetic changes in response to ELA and the way these modifications act or interact to predispose individuals to psychiatric disorders.

As technologies improve and their costs decrease, it will become feasible to combine multiple forms of high-throughput gene expression and epigenetic data to gain a more comprehensive view of the role of different epigenetic modifications in influencing the transcriptome. Though the relationship between different types of epigenetic modifications is not fully understood (Roadmap Epigenomics Consortium et al., 2015), it is likely that combining DNA methylation data with information regarding levels of specific histone modifications (another form of epigenetic alteration) could provide insight into potential functional roles of specific genomic regions. Such integrative analysis may reveal the downstream effects of DNA methylation changes associated with ELA. Additionally, it has become clear that there is an important relationship between genetic variation (i.e., single-nucleotide polymorphisms) and DNA methylation, and this represents a key aspect of the interaction between genetics and the environment (Gaunt et al., 2016). As such, incorporation of genetic information into future studies can be used to determine whether certain polymorphisms may be more vulnerable to changes in methylation patterns, thereby altering resilience to stress.

Newer technologies, such as the Illumina EPIC arrays, have greatly increased their coverage of non-traditional methylation sites, such as specific enhancers and open chromatin sites, DNase hypersensitive sites, and miRNA promoter regions. Furthermore, advances in cell sorting methods, as well as the collection of DNA from multiple tissue sources from the same individual, will improve our understanding of tissue-specific effects, and our ability to use peripheral samples for diagnostic purposes.

Despite the heterogeneous methodological approaches that have been applied when studying altered methylation patterns in individuals who have been exposed to ELA, the research conducted to date has given us insight into the biological mechanisms of resilience and vulnerability. Increasingly, we are identifying other mechanisms of epigenetic influences on gene expression, which illustrate the complexity of individual response to stress and behavioral regulation. The most effective interventions may therefore need to be tailored to individuals’ biological, experiential, and social contexts, as informed by the growing literature in the field. Ultimately, these studies could allow us to identify potential biomarkers for individuals who may benefit from early treatment intervention, and identify putative targets for novel therapeutics.
